# Thrombin-Free Fibrillogenesis and Gelation of Fibrinogen Triggered by Magnesium Sulfate

**DOI:** 10.3390/gels9110892

**Published:** 2023-11-11

**Authors:** Dominik Hense, Oliver I. Strube

**Affiliations:** Institute for Chemical Engineering, University of Innsbruck, A-6020 Innsbruck, Austria; dominik.hense@uibk.ac.at

**Keywords:** fibrinogen nanofibers, fibrillogenesis, hydrogels, self-assembly, magnesium, sulfate, binding sites

## Abstract

Self-assembly of the blood protein fibrinogen is a highly relevant topic in materials science and medical research. This originates from fibrinogen’s beneficial material properties such as cell interaction and biocompatibility. Within recent decades, several enzyme-free strategies to create fibers and hydrogels out of fibrinogen have been presented, broadening the spectrum of fibrinogen-based material enormously. Herein, we describe a further method to obtain such a material by adding specifically MgSO_4_ to fibrinogen. The key of this material is the combination of Mg^2+^ and a kosmotropic anion, for example sulfate or (hydrogen)phosphate. This effect is most likely related to occupancy of fibrinogen’s well-known binding sites for Mg^2+^, resulting in a significant increase in fiber yield and gel stability. Here, we shine light on the question of how electrostatic interactions via Mg^2+^ enhance fibrillogenesis and the gelation of fibrinogen and discuss first insights into the material’s properties.

## 1. Introduction

Using biopolymers for everyday applications has always been the subject of manifold research, and especially modern approaches focus on the search for sustainable materials based on renewable resources. One of the main reasons is that nature provides a broad variety of different materials, which are all optimized for their respective application. Mankind increasingly recognizes this high potential of biological materials, which has, for example, led to DNA origami [1], commercially available alginate-based wound dressings [2], as well as coatings and nanostructures with casein [3,4] or melanin [5,6].

One biological polymer of particular interest is the blood clotting protein fibrinogen. In an enzyme-catalyzed reaction, fibrinogen polymerizes to fibrin, a fibrous hydrogel designed for wound sealing. This network is especially designed for cell attachment, so newly growing tissue can adhere on fibrin [7]. Since it is comparatively uncomplicated to isolate fibrinogen and the required enzyme, thrombin, fibrin has gained high interest for manifold in vivo and in vitro applications ranging from biochemistry over materials science to medical applications. The most famous application is fibrin glue in surgery, which guarantees fast and uncomplicated wound healing [8,9,10,11,12]. Other applications include synthetic skin scaffolds in tissue engineering [13,14], drug delivery [15,16,17], cell delivery [18], and bio-printing [19,20]. In terms of cost and risk of thrombosis, it could be advantageous to use thrombin-free fibrinogen-based materials instead of fibrin. These two aspects actually resemble a noteworthy downside of this otherwise universally applicable material. The most essential properties of fibrin, cell-adhesion and biocompatibility, are already provided by its precursor fibrinogen [7], and fibrin is in this case responsible for providing an actual matrix in the form of a hydrogel. The goal to create inexpensive, thrombin-free alternatives to fibrin has led to various approaches to gel fibrinogen or to create fibers out of it. Many of those materials are designed for one distinct application and the following paragraphs shall give a brief overview over some advances in this field.

In general, fibrinogen-based materials can be categorized into four categories, dependent on their fibrous or gelled structure. Non-gelled and non-fibrous materials are frequently used as (nano-) particles in drug delivery and tissue engineering [21,22,23]. Another strategy is to lyophilize (native) fibrinogen from solution to obtain porous aerogels. These have shown high potential in animal experiments for in vivo bone repair [24]. This kind of materials is, however, comparatively less used than the gelled or fibrous alternatives.

Non-fibrous fibrinogen hydrogels can be obtained by several strategies. In its simplest form, fibrinogen is denatured by heat, which leads to a hydrogel [25]. Another method is to add a cross-linker to introduce covalent bonds, eventually leading to three-dimensional networks. This can be, for example, the enzyme factor XIII [26], a transglutaminase responsible for stabilizing fibrin networks. In this context, it was found that the addition of CaCl_2_ triggers a gelation of fibrinogen due to residual factor XIII in the delivered fibrinogen batches. Addition of the factor XIII inhibitor 2-iodoacetamide solved this issue and prevented gelation [27,28]. It was, however, explicitly ruled out that factor XIII triggers the formation of fibers in these experiments [29].

Also, the addition of specific salts can induce gelation. Steven et al. presented an early example of such effects by adding specific metal cations such as Fe^2+^, Ni^2+^, Cr^3+^, or Hg^2+^. Remarkably, neither Ca^2+^ nor Mg^2+^ induced any effects in this study [30].

Salt-induced approaches proved to be a feasible technique to create another type of fibrinogen-based material, which is mostly fibrous but non-gelled. The addition of specific salts under suited conditions triggers a self-assembly process, which leads to fibrous structures. Already in the 1980′s, Gollwitzer et al. observed fibrous aggregation of fibrinogen when it is dialyzed against buffers of low ionic strength [31]. Another method to obtain fibrinogen fibers is by adding HCl to a fibrinogen solution to reach a pH of 2.0. This leads to amyloid-like fibers. It is assumed that the self-assembly is mediated by monovalent metal cations such as Na^+^ and K^+^ [32]. The same working group established another similar technique to obtain fibrinogen fibers, which is by adding ethanol to a fibrinogen solution. Again, amyloidosis could be the reason for fibrillogenesis. Gels are, however, not accessible by these two approaches because the process has an upper limit of possible fibrinogen concentrations of 50 mg/L [33]. 

Self-assembly of fibrinogen is certainly not limited to processes in solution but can also be mediated by surfaces. Such techniques usually yield higher amounts of fibers. Recently, a salt-induced approach has been introduced, which requires the presence of monovalent cations such as Na^+^. Upon drying, large quantities of fibers emerge if at least 2 g/L fibrinogen are present [34,35]. On a side note, the requirement of a minimum fibrinogen concentration is a remarkable contrast to the previous Na^+^/K^+^-mediated fibrillogenesis, which has a comparatively low maximum concentration. Divalent cations have no effect in this specific experimental setup [36]. Another working group showed that fibrinogen can undergo fibrillogenesis on hydrophobic surfaces, even if no salts are present [37]. This effect is mediated by fibrinogen’s αC region, which reaches out into the surrounding area and connects to other fibrinogen molecules. This would not be possible on hydrophilic substrates since the (hydrophilic) αC region is in these cases responsible for adsorption on the support material and cannot reach neighboring molecules [38].

There are many more examples of how fibrinogen-based materials can be used as alternatives to fibrin or how (enzyme-free) self-assembly to fibers can succeed. Most of these approaches focus either on gelation or fibrillogenesis, but not many reports about thrombin-free fibrous hydrogels can be found. One remarkable approach to create such materials is based on fibrin protofibrils. In a first step, thrombin is added to a fibrinogen solution to cleave fibrinopeptides A and B. After a short incubation time, thrombin is immediately quenched so that only fibrinogen protofibrils remain. Cleavage of fibrinopeptides A and B has exposed additional binding sites for Ca^2+^ and Zn^2+^, respectively. After addition of the respective cation, a fibrous hydrogel forms from this solution of protofibrils. Although not entirely thrombin-free, the actual self-assembly is triggered by salts [39,40,41].

In our own previous research, we have established two completely thrombin-free and salt-induced approaches to create fibrous fibrinogen gels in solution. One of them relies on kosmotropic anions such as sulfate, (hydrogen)phosphate, or tartrate to create a gel-like fibrous material. This process, however, requires conditions close to fibrinogen’s solubility limit, i.e., temperatures of 5 °C, low overall salt concentration (<20 mmol/L), and a neutral to slightly acidic pH [42]. Due to this, the applicability of this method appears to be limited. The discovery of this phenomenon was closely connected to the formation of branched fibrinogen microgels, which emerge when lowering the ionic strength of a fibrinogen solution. Lower ionic strength led to a more rapid aggregation. This process was studied via time-resolved light scattering and atomic force microscopy [43].

Our second approach to create fibrous fibrinogen hydrogels solely requires Ca^2+^ ions. This process is much more robust and applicable regarding higher temperatures, salt concentrations, and pH, so that a comparatively high yield of fibrous gels is accessible [44]. It is not clear whether unintended cross-linking via factor XIII interferes with this process [29,45]; it is, however, most likely not the only origin of this phenomenon. The hypothesis arose that binding sites for Ca^2+^ (and Mg^2+^) contribute to this phenomenon in a not yet fully understood way. It is already known that fibrinogen can bind several Ca^2+^ and Mg^2+^ ions; the exact number, however, depends on the pH [46]. Especially, binding of Ca^2+^ has a remarkable influence on fibrinogen and fibrin. Known effects of Ca^2+^ binding are, e.g., increased resistance against thermal and acid denaturation. Additionally, fibrin becomes more resistant against plasmin degradation [47].

The focus of this work shall be to understand the function of binding sites even more; this time, however , with Mg^2+^ as cation. This will lead to another remarkable self-assembly of fibrinogen if suited counter anions are chosen. Potentially, the findings will contribute to a broader understanding of fibrinogen’s functions and features, lay the base for further mechanistic insights of fibrinogen self-assembly, and even help to develop a material close to natural fibrin.

## 2. Results and Discussion

### 2.1. Fibrillogenesis Triggered by MgSO_4_ and Comparison to Our Previously Introduced Processes

Previously, we introduced two rationales to induce fibrillogenesis of fibrinogen in solution to create “pseudo-fibrin”. One of them is driven by kosmotropic anions according to the Hofmeister series [42], while the second one solely requires Ca^2+^ [44]. The latter is significantly more potent than the anion-triggered approach and even yields fibrous hydrogels under physiological conditions. A brief summary of the two approaches is shown in Table 1. Already on first sight, significant differences between the two processes become apparent.

Since Ca^2+^ is such a potent trigger, the hypothesis emerged that the binding sites for Ca^2+^ are involved in the process. It is known that Mg^2+^ can bind to fibrinogen to a certain extent as well due to its net negative charge [46], so complementary experiments were performed with magnesium salts.

**Figure 1:**
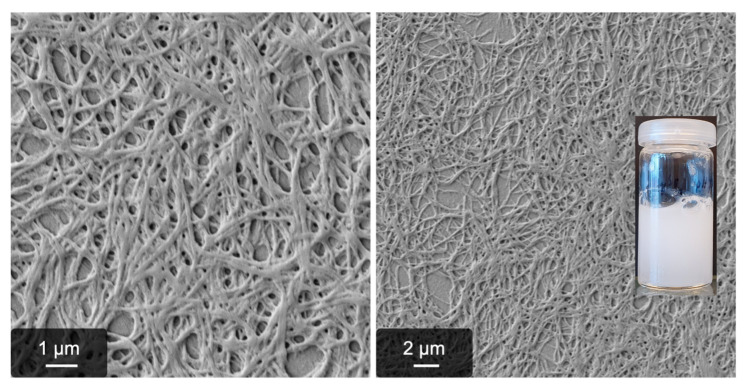
Impression of MgSO_4_-induced fibrinogen fibers after 24 h reaction time at 5 °C. Shown are SEM images of the fibers and a photograph of the sample.

As already described in our previous work [44], MgCl_2_ leads to a “semi-fibrous” material which is gel-like but contains mostly unstructured, precipitated fibrinogen. Now, to identify possible (disrupting) influences of the anion, two other magnesium salts were considered as triggers for fibrillogenesis. Again, all reaction conditions were kept identical to the previous process, i.e., a temperature of 5 °C, 15 mmol/L of the trigger salt, and pH 7.0. By using Mg(NO_3_)_2_ as a trigger, basically the same results were obtained as already known from MgCl_2_. With MgSO_4_, however, high amounts of fibers were reproducibly observed with scanning electron microscopy (Figure 1). As already done in previous experiments, thrombin catalysis was ruled out by addition of the inhibitor AEBSF and potential residuals of factor XIII were deactivated with iodoacetamide. This result is at first surprising since neither Mg^2+^ nor SO_4_^2-^ trigger such an intense self-assembly process on their own [42,44].

This finding raises two main questions which shall be addressed in the following sections. First, this effect shall be integrated into the context of our other salt-induced processes to create pseudo-fibrin (Table 1). The main goal is to identify whether we actually discovered a third salt-induced process or if this process is somehow linked to one of the two others. This shall first be accomplished by identifying commonalities and differences between these three processes. As shown in Table 1, relevant parameters for this comparison are possible reaction temperature, reaction times, salt concentrations, and pH. Second, the origin of this apparently specific “MgSO_4_ effect” shall be investigated. For this purpose, we will combine anion-induced pseudo-fibrin with occupancy of fibrinogen’s well-known binding sites for Mg^2+^.

**Figure 2:**
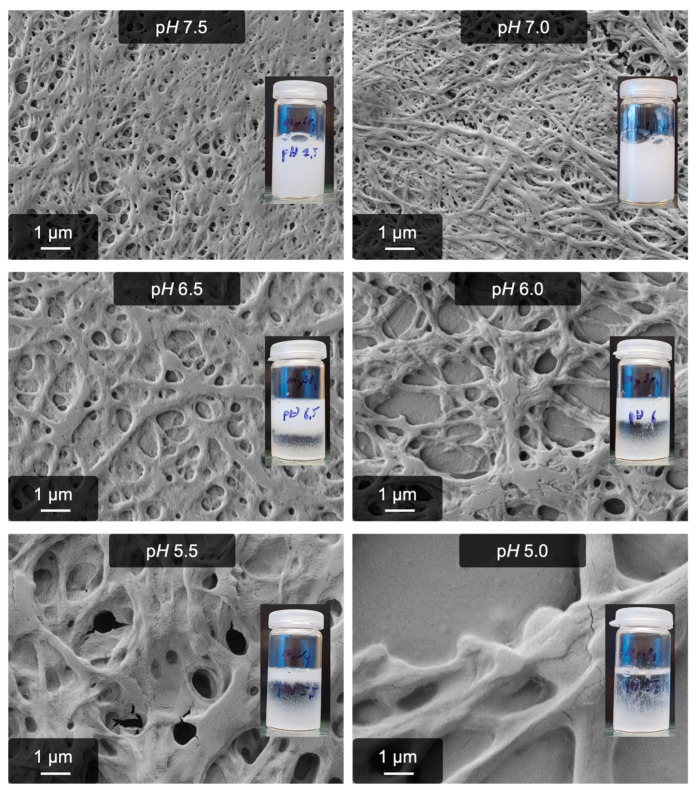
pH-dependency of MgSO_4_-induced pseudo-fibrin. Shown are SEM images and photographs of the corresponding samples.

As a first step, a pH series was performed. The insolubility of Mg(OH)_2_ makes it, however, impossible to investigate this process at pH > 7.5 since the 15 mmol/L Mg^2+^ interferes with the NaOH required to reach alkaline pH. As shown in Figure 2, the optimum pH for this process is 7.0–7.5, whereas acidic pH rapidly decreases fiber yield and gel stability. The results at pH ≤ 6.0 are similar to the ones obtained with Na_2_SO_4_, i.e., the anion-induced pseudo-fibrin formation [42]. Further commonalities were found regarding the temperature dependency. In both cases, values approaching physiological conditions slow down or even inhibit the process and neither fibers nor a gel are obtained. Actually, already prepared gels even dissolve at 37 °C. A subsequent re-gelation by cooling the samples to 5 °C is, however, not possible and leads to non-fibrous aggregates.

**Figure 3:**
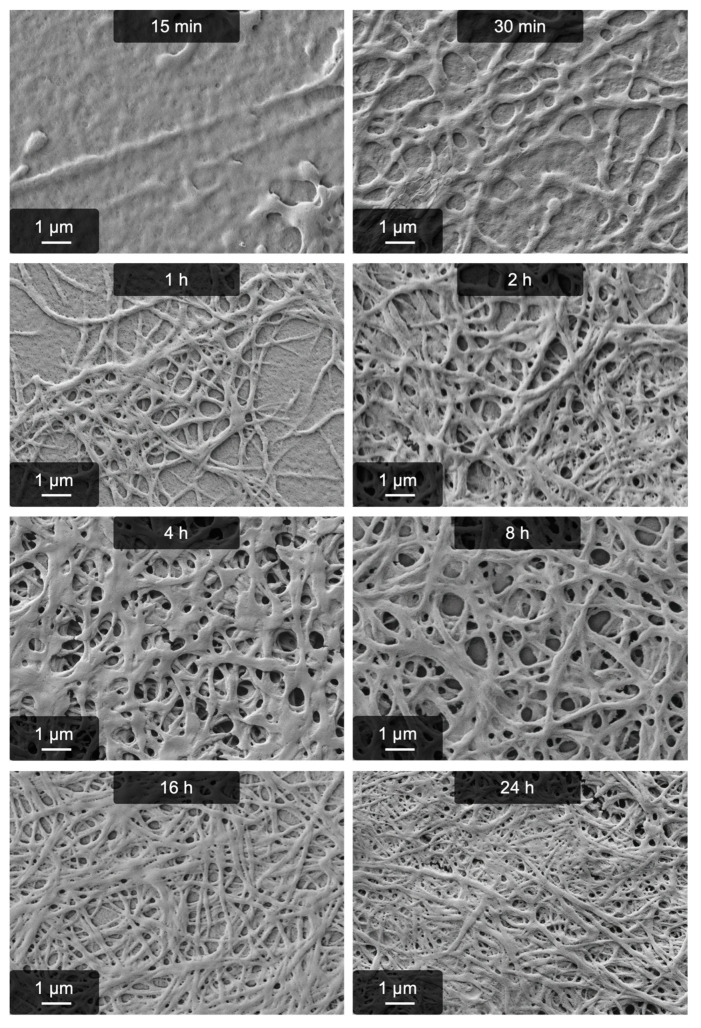
Fibrillogenesis induced by MgSO_4_ during the first 24 h after salt addition. It takes only 1 h until significant amounts of fibers have emerged and 2 h until the whole sample is covered in fibers.

An interesting difference to both other approaches to create pseudo-fibrin was found in the reaction time. SEM images of the “MgSO_4_ process” (Figure 3) do not show significant differences after 2 h anymore, indicating a comparatively fast process. Thrombin-induced fibrin formation is certainly much faster than 2 h; our two previously found salt-induced methods, however, require 16 h (with Ca^2+^) or 4 h (with kosmotropic anions) until they can be considered complete. On a side note, these SEM images rule out surface-induced fibrillogenesis because the chemical composition of the samples is identical in all cases. Otherwise, in the case of surface-induced effects, all samples should look identical.

**Figure 4:**
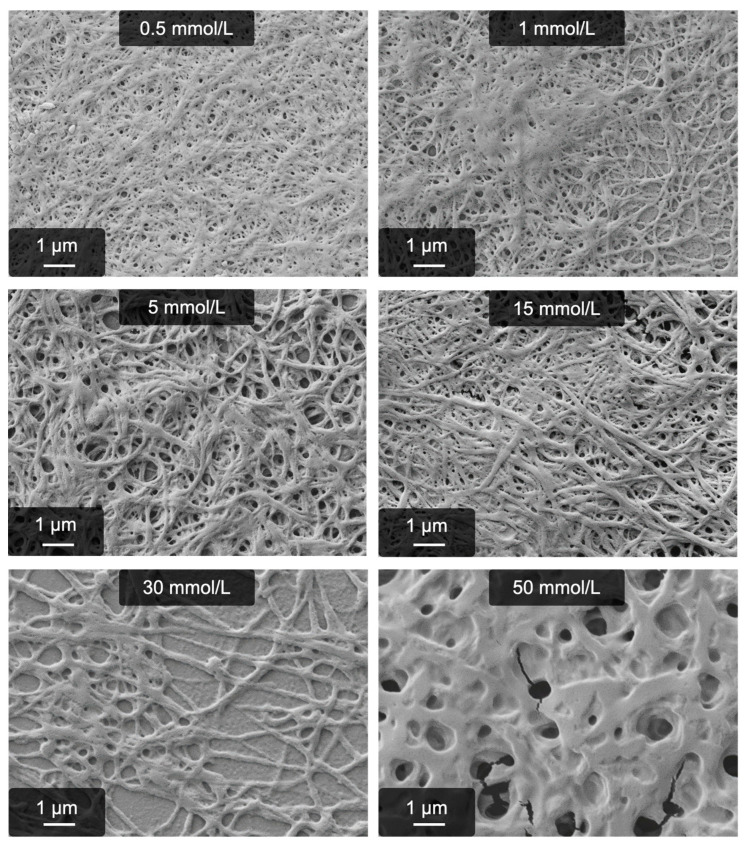
Concentration dependency of the MgSO_4_-induced process.

**Figure 5:**
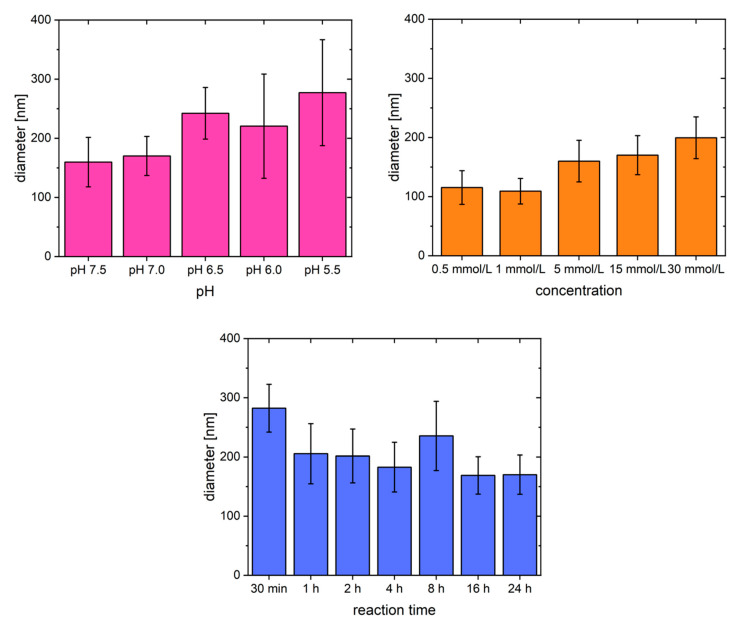
Comparison of average fiber diameters obtained by varying pH, MgSO_4_ concentration, and reaction time.

The final relevant parameter is the concentration range in which fibrillogenesis can successfully be triggered. As can be seen in Figure 4, even with 0.5 mmol/L MgSO_4_, significant amounts of fibers are obtained. These are rather thin compared to higher concentrations (a detailed analysis of fiber diameters is found in Figure 5). Below 0.5 mmol/L, fiber yield and gel stability rapidly decrease, although some fibers are still formed. At low concentrations, other effects have to be considered, which are discussed in more detail in Section 2.3. The upper limit of MgSO_4_ concentrations is around 50 mmol/L. At this concentration, no gelled (or even gel-like) material is obtained. Compared to our Ca^2+^-induced route, the range of possible MgSO_4_ concentrations is very similar (see Table 1). On the other hand, it is much broader compared to the anion route.

To obtain a more quantitative insight into MgSO_4_-triggered fibrillogenesis of fibrinogen, average fiber diameters were determined from the SEM images above. The results are shown in Figure 5 and raw data can be found in the supporting information (Tables S1–S3). In general, the fiber diameters mostly range from 100 to 250 nm. Even higher diameters of more than 300 nm are only obtained when the pH becomes acidic (pH < 6.0). However, the yield of actually fibrous structures rapidly decreases at these pH values and also the material itself cannot be considered as hydrogel anymore. Instead, unspecific aggregation of fibrinogen to non-fibrous precipitates becomes more pronounced.

Regarding the reaction time, all fiber diameters are very similar. They are in the range of 180 nm in all cases, although by tendency the diameter slightly decreases with increasing reaction time. The thickest structures were found early during the reaction, i.e., after 30 min. However, the corresponding SEM image shows that defined fibers have not yet emerged and some spherical structures are present. How these may be involved in fibrillogenesis cannot be determined yet, however, they lead to an apparent increase in fiber size.

Finally, the concentration dependency of the process represents a useful strategy to tailor the fiber diameter. With 0.5 or 1 mmol/L MgSO_4_, it is even possible to obtain fibers with a diameter <100 nm. The upper limit, achieved by varying the concentration, is approximately 200 nm (30 mmol/L MgSO_4_). Even higher salt concentrations eventually favor the dissolution of fibrinogen, competing with the gelation. This effect was also very prominent during our earlier approaches to obtain thrombin-free, fibrous hydrogels [42,44].

### 2.2. Rheological Characterization

As was already done in our previous studies, the material was characterized by means of rheology. Such measurements are important to estimate the material’s application potential as they indicate the mechanical properties of a hydrogel. Especially, storage modulus G’ and loss modulus G’’ provide useful information about the gel stability, degree of cross-linking, and reversibility of cross-linking [48].

**Figure 6:**
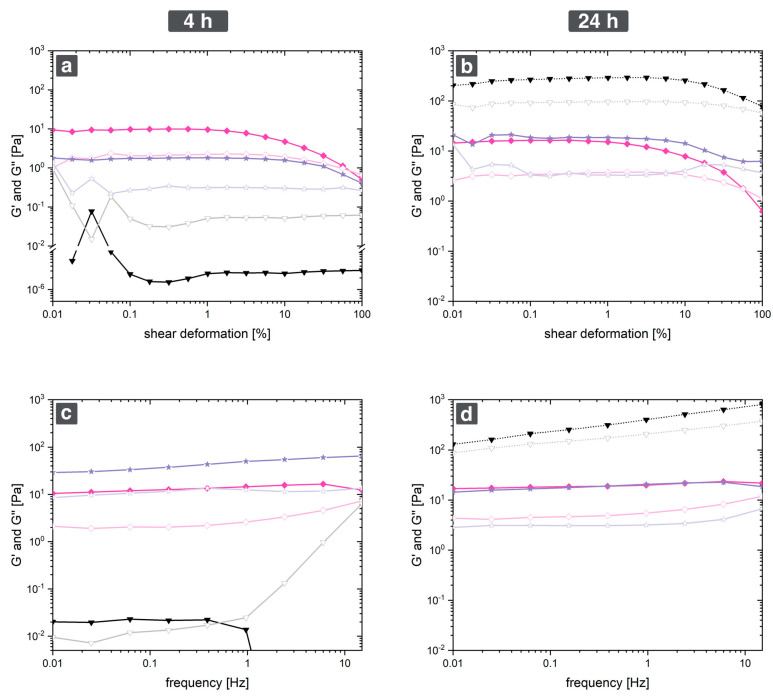
Rheologic characterization of MgSO_4_−induced pseudo−fibrin (◆) compared to fibrin (★) and native fibrinogen (▼). Shown are amplitude scans (**a**,**b**) and frequency scans (**c**,**d**) after 4 h and 24 h reaction time. Closed symbols represent G’ while open symbols represent G’’.

All measurements were performed after 4 h and 24 h reaction time. As references, pure fibrinogen in ultrapure water without salts and fibrin in phosphate-buffered saline were chosen. Figure 6 shows the results of an amplitude and frequency sweep for MgSO_4_-induced gels compared to native fibrinogen and fibrin. The first noteworthy aspect is the apparent rheological behavior of pure fibrinogen. This was solely dissolved in water and stored for 4 h (or 24 h, respectively) at 5 °C. After 4 h (figures a + c), this solution is barely measurable. However, after 24 h (figures b + d), it appears that this native fibrinogen is even more stable or stiffer as its storage modulus surpasses the one on fibrin by one order of magnitude. This is, however, only an artifact stemming from precipitation of fibrinogen. After 24 h, a dense, non-fibrous precipitate had formed on the bottom of the reaction vessel, which leads to the shown rheological behavior. The sample could not even be considered gel-like and the liquid phase was again barley measurable with the given setup. The high moduli most likely stem from the fact that this precipitate was comparatively dense. It consisted mostly of protein, while fibrin and pseudo-fibrin gels are mostly water.

The MgSO_4_-induced pseudo-fibrin sample and fibrin did not show any inhomogeneities (see also Figure 1 for an optical impression). Thus, the measurements do not contain such artifacts but show the actual behavior of the respective gel.

First, the results after the 4 h reaction time shall be discussed (figures a + c). Both materials show a pronounced linear viscoelastic regime in the amplitude tests. For fibrin, this regime lasts until a deformation of approximately 10%, whereas the MgSO_4_-induced gel already begins to collapse at 2% shear deformation. This indicates a lower stability of the MgSO_4_-induced gels compared to fibrin. Indeed, the curves of G’ and G’’ intersect at shear deformations of 60%, meaning that the material collapsed. Although G’ and G’’ of fibrin approach each other, an intersection was not found in the measured range. In the frequency scan, both materials remain nearly undamaged, at least up to 15 Hz. Another important aspect is the absolute value of G’ and G’’ to gain insights into material stiffness and, consequently, the degree of cross-linking. Here, the frequency and amplitude scan show different results. While fibrin’s G’ ranges at 2 Pa in the amplitude scan, it is about 12 Pa in the frequency scan. Regarding all three other measurements and the generally very fast reaction time of thrombin, the low values of 2 Pa for fibrin appear not reasonable. However, reproduction of the measurement with another sample yielded similar results, making it difficult to scientifically interpret this effect [49].

The results of rheological characterization after 24 h (figures b + d) are surprising. As already discussed, the reference of native fibrinogen formed a dense, non-fibrous precipitate at the bottom of the vessel, which explains the high values of G’ and G’’. For this reason, the focus shall again be the comparison of MgSO_4_-induced pseudo-fibrin and fibrin. Both materials now show very similar values of G’ and G’’, especially in the frequency scan. Still, the curves of G’ and G’’ intersect in the case of MgSO_4_-induced gels, meaning that the gels can even collapse after 24 h reaction time. The intersection is still reached at 60% shear deformation, indicating that the material does not differ too much compared to reaction times of only 4 h. Also, the only slightly increased absolute values of G’ and G’’ indicate that the process is nearly complete after 4 h reaction time.

In all cases, the measured values for fibrin are rather low compared to the literature values of ≈1000 Pa [49]. This observation is due to the low fibrinogen concentration of 5 g/L, which leads to more soft gels. Still, this concentration is required to obtain a suited reference material to MgSO_4_-induced pseudo-fibrin. This reference shows that MgSO_4_-induced pseudo-fibrin actually has similar rheologic behavior compared to fibrin, with the major difference being a shorter linear viscoelastic regime in the case of pseudo-fibrin. Both materials, fibrin and pseudo-fibrin, significantly differ from native fibrinogen. A summary of the most important information of this rheological comparison is provided in Table 2.

### 2.3. The Role of Binding Sites for Divalent Cations: Insights into the Mechanism

As already discussed above, we found several similarities between the “MgSO_4_ effect” and our two previously discovered approaches to create pseudo-fibrin. Based on these similarities, the hypothesis arose that all three routes are linked in some currently unidentified way instead of being three separate mechanisms to create a similar material.

We already know from our previous work that Na_2_SO_4_ induces (slight) gelation and fibrillogenesis as well, yielding a material with similar properties to the MgSO_4_-induced gel [42]. The now-observed higher fiber yield and shortened reaction time compared to the Na_2_SO_4_ route might therefore be an enhancement stemming from Mg^2+^ ions. Since also Ca^2+^ salts—independent of the anion—induce a highly effective gelation and fiber formation [44], we assume that the binding sites for Ca^2+^ and Mg^2+^ play a crucial role in the salt-induced gelation and fibrillogenesis of fibrinogen. For bovine fibrinogen, the actual number of binding sites varies with pH as most of them vanish under acidic conditions, due to protonation. They can also be distinguished in high-affinity binding sites (only suited for Ca^2+^) and low-affinity binding sites (suited for both, Mg^2+^ and Ca^2+^). Pioneering work on this field was performed by Marguerie et al. A list of the available binding sites at a given pH is shown in Table 3 [46]:

The low-affinity binding sites, which are the only ones suited for Mg^2+^, interestingly completely vanish at even slightly acidic pH due to protonation. This resembles an important analogy to the herein observed pH dependency (Figure 2). It is noteworthy that low-affinity binding sites originate from the net charge of fibrinogen, while the specific ones are due to chelate complexes. That means Ca^2+^ is actually incorporated into the molecule, whereas Mg^2+^ is not [46]. With these prerequisites, it is now possible to evaluate qualitatively whether binding sites are actually involved in any of our described self-assembly processes.

**Figure 7:**
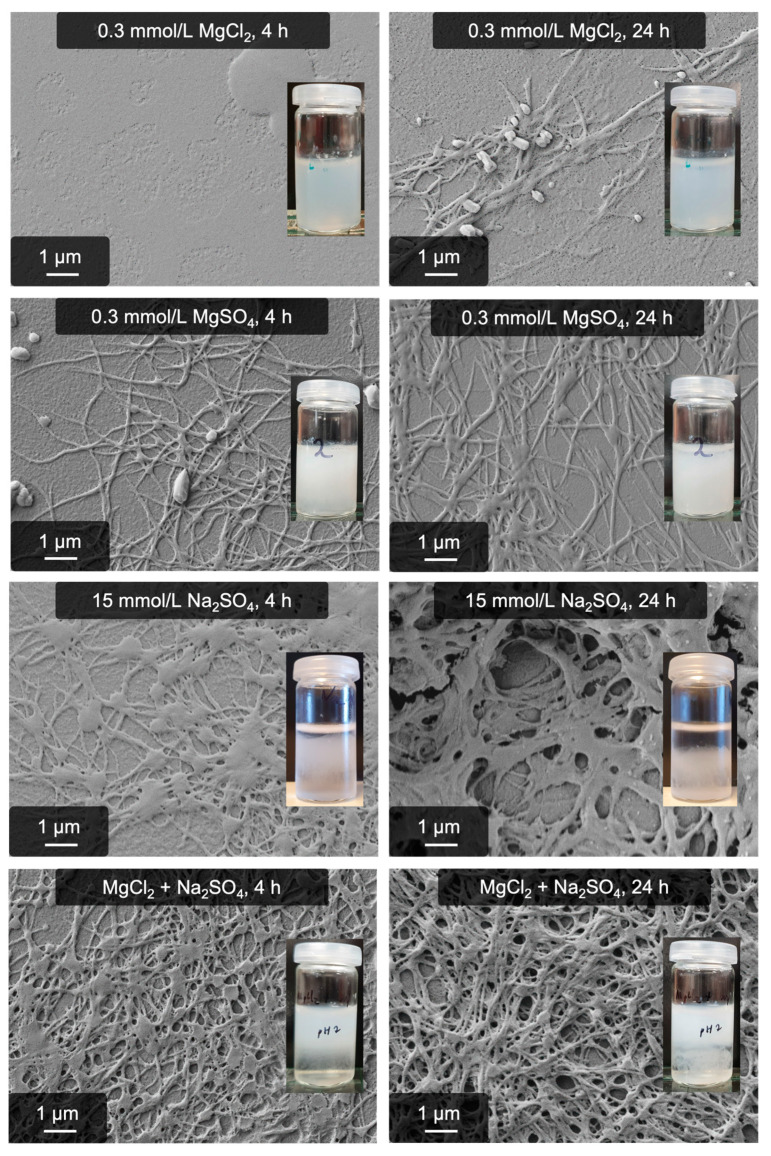
Influence of binding-site occupancy in pseudo-fibrin formation. Shown are samples with 0.3 mmol/L MgCl_2_ (reference), 0.3 mmol/L MgSO_4_, 15 mmol/L Na_2_SO_4_ as introduced in our previous work, and the 15 mmol/L Na_2_SO_4_ with occupancy of binding sites by 0.3 mmol/L MgCl_2_.

The rationale is now to combine Mg^2+^, i.e., occupancy of low-affinity binding sites, with a kosmotropic anion. This can be, for example, sulfate or (hydrogen)phosphate. Comparison of the samples shows whether binding site occupancy with Mg^2+^ enhances the fibrillogenesis triggered by kosmotropic anions, i.e., Na_2_SO_4_. Statistically, 0.3 mmol/L MgCl_2_ is required to saturate all binding sites in the used 5 g/L fibrinogen solution. The following Table 4 gives an overview of which experiments were performed to elucidate the influence of binding site occupancy on pseudo-fibrin formation:

All samples were stored at 5 °C and inspected after 4 h and 24 h. The results were remarkable: After four hours, the reference samples, containing only 0.3 mmol/L MgCl_2_ or MgSO_4_, were slightly cloudy, not gel-like, and consisted of only a few fibers. The Mg-free pseudo-fibrin consisted of some fibers and showed the typical medusa-shaped gel-like material. The sample combining Na_2_SO_4_ with binding site occupancy, however, evolved into a drastically different material; the material was remarkably more stable than the reference. These findings are visualized in Figure 7.

Combining binding site occupancy with a different kosmotropic anion, for example (hydrogen)phosphate, qualitatively yields the same result. The significant increase in fiber yield is solely attributed to binding site occupancy, which enables valuable insights into the mechanism(s) of fibrillogenesis. They also explain why specifically MgSO_4_ triggers a strong fibrillogenesis. In contrast to other Mg^2+^ salts, sulfate as a kosmotropic component triggers the anion process while Mg^2+^ occupies the binding sites of fibrinogen to enhance the process. Other Mg^2+^ salts such as MgCl_2_ and Mg(NO_3_)_2_ do not induce this effect since the anion is too chaotropic. As a result, the MgSO_4_-induced process can be described as an enhanced variant of the previously found anion-driven mechanism, which explains the numerous commonalities between both processes.

**Figure 8:**
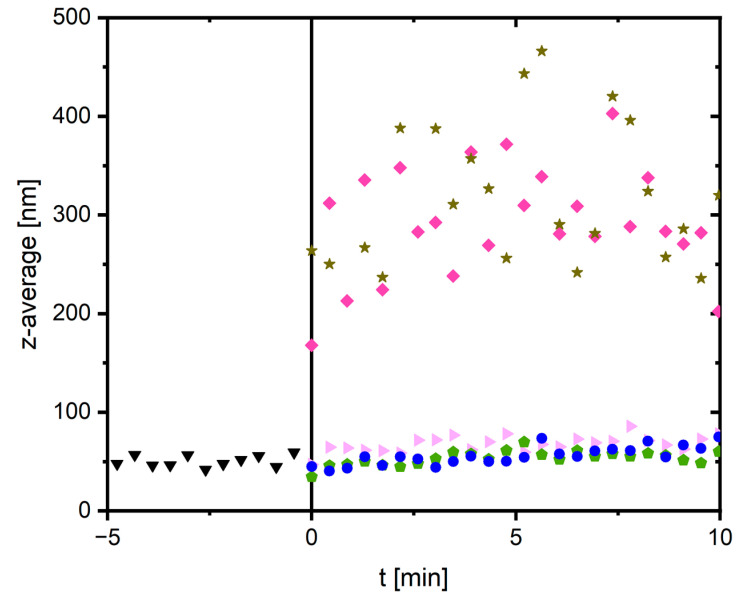
Time-resolved dynamic light scattering measurement to study the influence of binding site occupancy and addition of kosmotropic anions. Before addition of any salt, the pure fibrinogen solution was measured to guarantee its stability. This is shown in the time frame from −5 to 0 min (▼). At t = 0 min, different salts were added; 0.3 mmol/L MgCl_2_ (►) or 0.3 mmol/L MgSO_4_ (⬟) for binding site occupancy have nearly no effect. Adding 15 mmol/L sodium sulfate induces very slight aggregation, but the z-average increased by <10 nm (●). As a reference, 15 mmol/L MgSO_4_ induces strong aggregation (◆). Combining 0.3 mmol/L MgCl_2_ for binding site occupancy with a kosmotropic anion, in this case 15 mmol/L sodium sulfate, leads to a synergy and an increased tendency to form aggregates compared to either of the single components (★).

To quantify these findings, time-resolved dynamic light scattering was performed on analogous samples. The results are shown in Figure 8 and a reproduction experiment can be found in the supporting information (Figure S1). As a first step, the pure fibrinogen stock solution was measured for 5 min to guarantee that it does not aggregate by itself. Adding 0.3 mmol/L MgCl_2_ or MgSO_4_ induces only minimal aggregation of fibrinogen as the particle size (expressed as z-average) remains nearly constant during the measured time frame. Adding sodium sulfate to the fibrinogen solution should trigger anion-induced pseudo-fibrin formation; however, the size remains nearly constant. This effect is most likely attributed to the temperature of 10 °C, which is higher than usually applied to trigger this process. Ideally, aggregation would by triggered at 5 °C, as shown in our previous work [42]. It can already be seen from the photographs in Figure 7 that the addition of Na_2_SO_4_ leads to aggregation of fibrinogen at lower temperatures. Lowering the temperature even further was, however, not possible because of condensed water on the light scattering cuvette. Despite this difference to our previous work, the most interesting phenomena occur when Mg^2+^ is introduced into the process. Adding MgSO_4_ leads to a strong and rapid aggregation with highly fluctuating data points between 200 and 400 nm. These fluctuations are artifacts stemming from the large aggregates. Finally, combining traces of MgCl_2_, i.e., 0.3 mmol/L, with 15 mmol/L sodium sulfate, again leads to a strong and fast aggregation. The difference to pure sodium sulfate and pure MgCl_2_ is apparent. As already observed via SEM, combining binding site occupancy with a kosmotropic anion triggers a synergistic effect.

Mechanistically, we hypothesize that the electrostatic interactions between negatively charged fibrinogen molecules and Mg^2+^ cations result in an enhanced tendency to form aggregates. In fact, theoretical work considering the charge distribution in the fibrinogen molecule showed that the outer domains of the protein, the D domains, are negatively charged at physiological pH, making electrostatic interactions with Mg^2+^ at least plausible [50]. How the kosmotropic anions “align” the molecules to form fibers is, however, not yet clear but most likely obeys the same principles as in our purely anion-driven process [42]. Additional insight might be obtained in future studies via, e.g., FTIR measurements (to monitor the protein folding) or combined AFM/light scattering techniques to analyze the growth of fibers [43].

It is important to note that the exact number of occupied binding sites is not known for the described experiments. The amount of Mg^2+^ ions required for a certain degree of occupancy varies with fibrinogen concentration and always demands an excess of ions. In a 5 g/L solution of fibrinogen at pH 7.0, approximately 0.3 mmol/L binding sites are available. Complete occupancy is, however, not reached when adding this concentration of Mg^2+^. Based on literature reports, an occupancy of approximately 40–50% is estimated for our described experiments [46]. The low applied concentration of MgCl_2_ is, however, necessary to not induce fibrillogenesis on its own because MgSO_4_ (or MgHPO_4_) is formed during the reaction. As seen before, even 0.5 mmol/L MgSO_4_ is sufficient to trigger fibrillogenesis, so this concentration must not be surpassed.

On a side note, analogous experiments using Ca^2+^ salts are not comparable since Ca^2+^ first occupies all three high-affinity binding sites. Occupancy of both types of binding site is most likely the reason why Ca^2+^ triggers the, for now, most intense fibrillogenesis and gelation. In fact, several literature reports describe a re-folding of the protein in the presence of Ca^2+^ [47,51], which potentially favors fibrous aggregation independent of “structure providing” kosmotropic anions. Such investigations, however, go beyond the scope of this work.

## 3. Conclusions

Specific magnesium salts, i.e., those with kosmotropic anions, trigger an intense fibrillogenesis and gelation of fibrinogen. This process is purely salt-induced and does not involve any contributions from thrombin or factor XIII. Similar to our previously introduced anion-triggered and Ca^2+^-induced self-assembly processes, fibrillogenesis requires low overall ion concentrations and low temperatures. Due to the similarities to both of our already established approaches, the hypothesis arose that Mg^2+^ salts fill the gap between the two. Indeed, there is evidence that Mg^2+^ electrostatically binds to fibrinogen and acts as “bridge” between the molecules. Suited kosmotropic anions such as sulfate and (hydrogen)phosphate on the other hand are then responsible for the actual fiber formation, as described in previous publications. Mixing slight amounts of MgCl_2_ for binding site occupancy with Na_2_SO_4_ leads to significantly increased fiber yield and gel stability compared to the procedure without Mg^2+^. This finding can even be extended by combining Mg^2+^ and kosmotropic anions in one salt: MgSO_4_ triggers an intense fibrillogenesis in a simple and cost-efficient way. On the other hand, MgCl_2_ itself and Mg(NO_3_)_2_ result in unspecific precipitation due to the absence of a kosmotropic anion. These MgSO_4_-induced gels were characterized by means of pH, temperature, and concentration dependency. Additional rheologic measurements confirmed the increased stability of the gels.

Electrostatic bridging of the negatively charged fibrinogen molecules as the main reason for magnesium’s enhancing effect is strongly supported by the combined results. A quantitative final proof of this hypothesis is, however, still pending. Promising approaches to study the mechanistic details are FTIR studies combined with high-resolution microscopy and time-resolved light scattering. While these issues will be addressed in future studies, the current results already bring significant new insights into fibrinogen’s well-known binding sites for divalent cations and how they can promote enzyme-free fibrillogenesis and gelation. Potentially, the results even contribute to developing an inexpensive, fibrin-like material for use in medical contexts such as tissue engineering, drug delivery, and bioprinting.

## 4. Materials and Methods

### 4.1. Materials

Bovine fibrinogen (≥95% protein of which ≥95% is clottable) was obtained from Merck and used without further purification. Thrombin (45 U/mg solid) was purchased from Sigma Aldrich. 4-(2-aminoethyl)benzenesulfonyl fluoride hydrochloride (AEBSF; purity ≥ 98%) stems from Carl Roth. 2-iodoacetamide (purity 98%) stems from thermo scientific. Experiments were carried out in water of HPLC grade (specific conductivity max. 1 μS/cm) purchased from VWR. All other chemicals, specifically the used salts, were of at least 98% purity and purchased from the usual suppliers.

### 4.2. Production of Hydrogels

To prepare MgSO_4_ stock solutions, the respective amount of salt was weighed in and ultrapure water was added to reach a defined concentration. The pH was adjusted to 7.0. In general, the concentration of all stock solutions was 500 mmol/L except for the ones used for binding site occupancy (see Section 4.4).

Fibrinogen solutions with a concentration of 5 g/L were prepared by suspending the protein in 5 °C cold ultrapure water and adjusting the pH to 7.0 with 0.1 M NaOH. The solution was stirred at 300 rpm for 10 min until all fibrinogen was dissolved.

To trigger fibrinogen self-assembly, 120 μL of the 500 mmol/L MgSO_4_ solution was added fast. During this step, stirring was strictly avoided. The final salt concentration in the reaction mixture was 15 mmol/L. For experiments using different salt concentrations, the added volume of the salt stock solution was adjusted. If not stated differently, the reaction mixture was stored for 24 h at 5 °C.

The procedure to perform experiments at different pH was similar. The required amount of NaOH/HCl to reach the respective pH was in all cases added together with the MgSO_4_ solution. Before salt addition, the fibrinogen solution still had a pH of 7.0.

To prepare samples for scanning electron microscopy, a 12 × 12 mm microscopy cover slide was used. This glass side was held for 5 min into the gel, and then it was removed and dried overnight at room temperature and ambient pressure.

### 4.3. Enzyme Inhibition

To rule out thrombin catalysis, 0.5 mmol/L of AEBSF was added to the reaction mixture prior to MgSO_4_.

To rule out factor XIII catalysis, at first a fresh stock solution of 2-iodoacetamide was prepared prior to each respective experiment. The concentration of this solution was 1 mmol/L. Then, 0.1 mmol/L was added to the reaction mixture prior to MgSO_4_.

Both inhibitors, AEBS and 2-iodoacetamide, were incubated for 10 min at 5 °C. Afterwards, 15 mmol/L MgSO_4_ (pH 7.0) was added and the reaction mixture was stored for 24 h at 5 °C.

### 4.4. Occupancy of Binding Sites with 0.3 mmol/L Mg^2+^

Two fibrinogen solutions were prepared as described before. Prior to addition of any trigger salt solution, 0.3 mmol/L MgCl_2_ solution (corresponding to 2.4 µL of a 500 mmol/L MgCl_2_ stock solution) was added and distributed in the fibrinogen solutions for 5 min. One sample, serving as reference, was stored for 24 h at 5 °C after this step. To the other sample, 120 µL of a 500 mmol/L sodium sulfate solution was added fast but without stirring, corresponding to 15 mmol/L. Then, the sample was also stored for 24 h at 5 °C.

To vary the required kosmotropic anion for pseudo-fibrin formation, this experiment was repeated with 15 mmol/L sodium phosphate buffer (pH 7.0) instead of Na_2_SO_4_.

An additional reference was prepared analogously by distributing 0.3 mmol/L MgSO_4_ instead of MgCl_2_ in the fibrinogen solution.

### 4.5. Scanning Electron Microscopy (SEM) and Image Analysis

Scanning electron microscopy was performed with a ZEISS “Neon 40”. Topographic information was collected using an SE2 detector. Additional information on material contrast was collected using an InLens detector. The acceleration voltage was 2 kV in all cases.

Samples of fibrinogen hydrogels were prepared as described earlier. Briefly, a 12 × 12 mm glass slide was used to extract some of the material. After drying the samples overnight at room temperature and ambient pressure, they were sputtered with a 3 nm thick layer of Au/Pd alloy.

Fiber diameters at ten different spots were measured using GIMP. The diameter (in pixels) was then converted into a length using the given scale bar.

### 4.6. Rheology

Rheological properties were monitored using an Anton Paar MCR 302 with a cone/plate setup. In the first step, an amplitude scan was performed to identify the linear viscoelastic regime. Then, a frequency sweep from 0.01 Hz to 15 Hz was performed at 1% shear deformation.

Pseudo-fibrin hydrogels were prepared as described earlier (5 mg/mL protein, pH 7.0, 15 mmol/L MgSO_4_, 4 or 24 h at 5 °C). A reference sample containing only fibrinogen and no additional salts/enzymes was prepared analogously to the pseudo-fibrin samples. Before the actual measurement, the device was equilibrated at 5 °C to not induce unintended dissolution of the gels.

Fibrin references were prepared by weighing 5 mg/mL fibrinogen in freshly prepared PBS at room temperature (25 °C). Clotting was induced by adding 5 U/mL thrombin. This procedure differs from the setup required for pseudo-fibrin formation by means of temperature, salt content, and pH. The reason is that these conditions resemble a more practical and common way to create fibrin so that a reasonable reference material is obtained. The measurement was also conducted at 25 °C instead of 5 °C.

Since rheologic measurements require undamaged samples, all described samples were prepared twice.

### 4.7. Dynamic Light Scattering to Study Binding Site Occupancy

Time-resolved dynamic light scattering was performed using a Malvern ZetaSizer Ultra (Malvern, UK). A 0.5 g/L stock solution of fibrinogen was prepared by weighing in 4 mg fibrinogen and adding 8 mL ultrapure water. Then, 4 µL of 0.1 mol/L NaOH was added to dissolve fibrinogen. The mixture was stirred for 10 min at 25 °C with 300 rpm. For each measurement, 1 mL of the stock solution was filtered into a single-use polystyrene cuvette using a 450 nm PES membrane. Table 5 lists the volumes of the given salt stock solution, which were added to 1 mL fibrinogen stock solution:

The cell housing was tempered to 10 °C. All samples were equilibrated for 30 s at this temperature. The actual measurement of each sample lasted 10 min. The final fibrinogen concentration was 0.25 g/L in all cases except for the measurement, which contained 0.5 g/L. For data evaluation, the hydrodynamic diameters measured from the detector at an angle of 12.8° were expressed as z-average. A refractive index of fibrinogen of 1.45 was assumed for all measurements.

## Figures and Tables

**Table 1:** Summary of our two previously introduced approaches to create “pseudo-fibrin” via salt-induced self-assembly.

	Anion-Induced Pseudo-Fibrin	Ca^2+^-Induced Pseudo-Fibrin
Triggers	Kosmotropic anions (sodium phosphate, sodium sulfate, …)	Ca^2+^ salts independent of the anion
Fiber yield, gel stability	Medium fiber yield, low stability	High fiber yield, high stability
Temperatures	5–8 °C (irreversible dissolution at higher temperatures)	5–37 °C
Trigger concentrations	5–20 mmol/L	1–30 mmol/L
pH range	6.5–7.5	7.0–9.5
Reaction time	4 h	16 h

**Table 2:** Summary of the rheologic properties of MgSO_4_-induced pseudo-fibrin, fibrin, and native fibrinogen. These data are based on the frequency scans.

	Pseudo-Fibrin	Fibrin	Fibrinogen
G’ after 4 h [Pa]	10	2	0.02
G’ after 24 h [Pa]	15	20	No reliable information
Crossover after 4 h [Hz]	None observed	None observed	0.5
Crossover after 24 h [Hz]	None observed	None observed	No reliable information

**Table 3:** Number of high- and low-affinity binding sites for Ca^2+^ and Mg^2+^ according to Marguerie et al. [46] These results are valid for bovine fibrinogen.

pH	Number of High Affinity Binding Sites (Only for Ca^2+^)	Number of Low Affinity Binding Sites (for Ca^2+^ and Mg^2+^)
5.5	2	0
6.0	2	0
7.5	3	14–16
8.5	3	10–12
9.0	3	10–12

**Table 4:** Description of experiments and references to identify the influence of binding site occupancy on anion-induced pseudo-fibrin.

Nr.	Salt Addition	Purpose
1	0.3 mmol/L MgCl_2_	Saturating all binding sites with Mg^2+^
2	15 mmol/L Na_2_SO_4_	Triggering the anion-induced pathway to create pseudo-fibrin
3	0.3 mmol/L MgCl_2_ + 15 mmol/L Na_2_SO_4_	Combining the anion process with binding site occupancy
4	0.3 mmol/L MgSO_4_	Reference, since #3 yields 0.3 mmol/L MgSO_4_

**Table 5:** Description of sample preparation to monitor the influence of binding site occupancy on anion-induced pseudo-fibrin formation.

Nr.	Sample	Addition of Stock Solution
1	Reference of pure fibrinogen	---
2	Aggregation triggered by MgSO_4_	1 mL of a 30 mmol/L MgSO_4_ solution
3	Anion-induced pseudo-fibrin with 15 mmol/L Na_2_SO_4_	1 mL of a 30 mmol/L sodium sulfate solution
4	Binding site occupancy with 0.3 mmol/L MgSO_4_	1 mL of a 0.6 mmol/L MgSO_4_ solution
5	Binding site occupancy with 0.3 mmol/L MgCl_2_	1 mL of a 0.6 mmol/L MgCl_2_ solution
6	Anion-induced pseudo-fibrin with binding site occupancy (sodium sulfate was added to sample 5)	61.9 µL of a Na_2_SO_4_ solution added to sample 5

## Data Availability

Data are contained within the article and Supplementary Materials.

## Supplementary Materials

The following supporting information can be downloaded at: https://www.mdpi.com/article/10.3390/gels9110892/s1, Table S1: Average fiber diameter at different pH. Table S2: Average fiber diameter at different MgSO4 concentrations. Table S3: Average fiber diameter at different reaction times. Figure S1: Reproduction of dynamic light scattering measurements.
