# Supplementary material for: Thrombin-Free Fibrillogenesis and Gelation of Fibrinogen Triggered by Magnesium Sulfate
Source: Gels. 2023 Nov 11;9(11):892. doi: 10.3390/gels9110892 (PMC10671114; doi:10.3390/gels9110892)
Supplement: Supplementary file 1 [file gels-09-00892-s001.zip › gels-2721229-supplementary.pdf]

Thrombin-free Fibrillogenesis and Gelation of Fibrinogen triggered by Magnesium Sulfate

Dominik Hense[1] and Oliver I. Strube[1]*

[1] Institute for Chemical Engineering, University of Innsbruck, Austria
* Correspondence: oliver.strube@uibk.ac.at, phone: +43 512 507 553 00

Table S1. Average fiber diameter at different pH.

pH	7.5	7.0	6.5	6.0	5.5
diameter [nm]	159.7 ± 41.8	170.0 ± 33.0	242.2 ± 43.7	220.5 ± 88.1	277.0 ± 89.6

Table S2. Average fiber diameter at different $MgSO_4$ concentrations.

$MgSO_4$ concentration [mmol/L]	0.5	1	5	15	30
diameter [nm]	115.4 ± 28.5	109.2 ± 21.6	160.0 ± 35.1	170.0 ± 33.0	199.5 ± 35.3

Table S3. Average fiber diameter at different reaction times.

reaction time [h]	0.5	1	2	4	8	16	24
diameter [nm]	282.2 ± 40.3	205.4 ± 50.6	201.6 ± 45.3	182.7 ± 41.9	235.4 ± 58.4	168.9 ± 31.6	170.0 ± 33.0

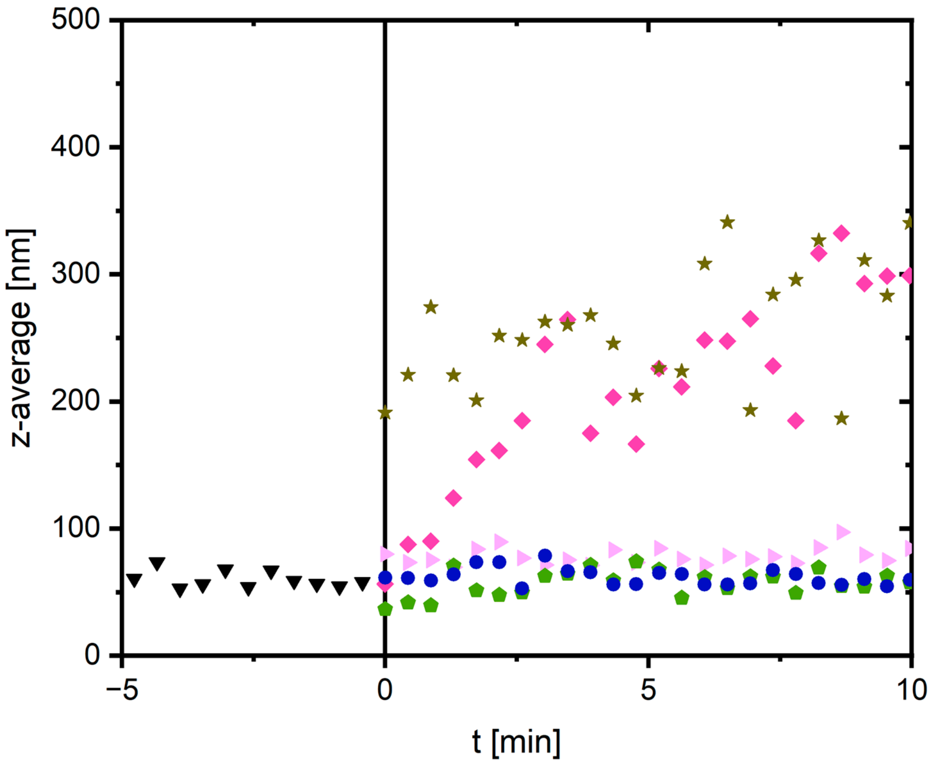

Figure S4. Reproduction of time-resolved dynamic light scattering measurement to study the influence of binding site occupancy and addition of kosmotropic anions. Before addition of any salt, the pure fibrinogen solution was measured to guarantee its stability. This is shown in the time frame

from -5 to 0 min (▼). At t = 0 min, different salts were added. 0.3 mmol/L $MgCl_2$ (▸) or 0.3 mmol/L $MgSO_4$ (⬟) for binding site occupancy have nearly no effect. Adding 15 mmol/L sodium sulfate induces very slight aggregation, but the z-average increased by < 10 nm (●). As a reference, 15 mmol/L $MgSO_4$ induce strong aggregation (◆). Combining 0.3 mmol/L $MgCl_2$ for binding site occupancy with a kosmotropic anion, in this case 15 mmol/L sodium sulfate, leads to a synergy and an increased tendency to form aggregates compared to either of the single components (★).
